# Beyond Money: Conscientious Objection in Medicine as a Conflict of Interests

**DOI:** 10.1007/s11673-020-09976-9

**Published:** 2020-05-12

**Authors:** Alberto Giubilini, Julian Savulescu

**Affiliations:** 1 Wellcome Centre for Ethics and Humanities and Oxford Uehiro Centre for Practical Ethics, University of Oxford, 16-17 St Ebbes Street, Littlegate House, Oxford, OX1 1PT UK; 2 Oxford Uehiro Centre for Practical Ethics and Wellcome Centre for Ethics and Humanities, University of Oxford, 16-17 St Ebbes Street, Littlegate House, Oxford, OX1 1PT UK; 3 Melbourne Law School, University of Melbourne, Melbourne, Australia

**Keywords:** Conflicts of interests, Conscientious objection, Professionalism, Conscience

## Abstract

Conflict of interests (COIs) in medicine are typically taken to be *financial* in nature: it is often assumed that a COI occurs when a healthcare practitioner’s financial interest conflicts with patients’ interests, public health interests, or professional obligations more generally. Even when non-financial COIs are acknowledged, ethical concerns are almost exclusively reserved for financial COIs. However, the notion of “interests” cannot be reduced to its financial component. Individuals in general, and medical professionals in particular, have different types of interests, many of which are non-financial in nature but can still conflict with professional obligations. The debate about healthcare delivery has largely overlooked this broader notion of interests. Here, we will focus on health practitioners’ moral or religious values as particular types of personal interests involved in healthcare delivery that can generate COIs and on conscientious objection in healthcare as the expression of a particular type of COI. We argue that, in the healthcare context, the COIs generated by interests of conscience can be as ethically problematic, and therefore should be treated in the same way, as financial COIs.

## Scenarios

### Consider the following scenarios

#### Antibiotics

John and Mary have a viral infection and both think, mistakenly, that they need antibiotics. In many parts of the world, clinically inappropriate antibiotic prescription is a widespread practice among physicians, who often give in to patients’ unjustified requests (Van der Velden et al. 2013; O’Neill 2016). This practice is a major contributing factor to antibiotic resistance, one of the most significant public health emergencies of our times. John requests a prescription for antibiotics from his doctor. His doctor regularly receives gifts and conference invitations (including hospitality in luxury hotels) from a major pharmaceutical company that produces antibiotics. Gifts and other direct and indirect payments to physicians are common practice among pharmaceutical companies. For instance, in the United States between August 2013 and December 2014, 1630 pharmaceutical companies spent $3.53 billion on direct and indirect general (non-research related) payments to about 700,000 health professionals to promote their drugs (Greenway and Ross 2017). Mary also asks her doctor for a prescription for antibiotics. Her doctor’s mother recently died of an antibiotic-resistant infection, which would have been treatable if antibiotic resistance had not developed so quickly in recent years. John’s doctor prescribes the requested unnecessary antibiotics, and Mary’s doctor refuses to prescribe them. We do not know whether the first doctor’s decision to prescribe antibiotics was influenced by the payments she received, nor whether the second doctor’s decision not to prescribe antibiotics was influenced by her mother’s death. But we do know that gifts from pharmaceutical companies to physicians are associated with a greater likelihood of prescribing those companies’ promoted drugs (Fleischman et al. 2016; DeJong et al. 2016).

#### Vaccination

Mark and Helen are parents who are hesitant about vaccinating their respective male children against human papilloma virus (HPV), a sexually transmitted disease which significantly increases the chances of developing cervical cancer and, to a lesser degree, anal, head, and neck cancer. Whilst both sexes are affected, HPV increases the risk of cancer in females to a greater extent than males. Following a widespread recent trend (Kmietowicz 2018), Mark and Helen’s local authority has extended HPV vaccine subsidization to male children, thus modifying a previous policy that subsidized female HPV vaccination only. The parents consult their doctors for advice on whether or not to vaccinate their male children. Although current best practice guidance is to vaccinate both males and females, doctors are still less likely to recommend the HPV vaccine to males than to females (Beavis et al. 2018). Mark’s doctor, very much like John’s doctor in the antibiotics scenario, receives gifts from a pharmaceutical company that produces the HPV vaccine. Helen’s doctor is a feminist, and she thinks that the subsidizing or recommending the HPV vaccine solely to females unfairly places the burden of responsibility for the prevention of the sexual transmission of HPV onto females. Mark and Helen’s doctors both provide the relevant information and spend time persuading them to administer the vaccine to their respective male children. We do not know whether, and if so to what extent, the financial interests and the ethical views of the doctors influenced their consultations. As a result of the consultation, both parents decide to vaccinate their male children.

#### Abortion

Jane and Joanna are pregnant and, due to serious medical complications, both need an abortion, which is legal in the country where they live. Joanna is in a hospital, and the doctor who is in charge of her care is a committed Catholic. Religious opposition to abortion among physicians is very widespread and typically protected by the law in the form of various “conscientious objection” policies. In certain countries, the rate of conscientious objection among physicians is so high that it can jeopardize timely and safe access to legal abortion. For instance, in Italy the rate of gynaecologists who conscientiously object to abortion is consistently above 70 per cent (with peaks of over 80 per cent in certain regions), and the law allows those with a conscientious objection to refuse to perform abortion (Minerva 2015). In Poland, women have died due to their doctors refusing to perform an abortion that would have been life-saving (Minerva 2017). Jane is in the same situation as Joanna, but her doctor is a feminist, and he strongly believes that, quite apart from his professional obligations, women have an absolute moral right to decide whether or not to terminate a pregnancy. Thus, both doctors have personal religious or moral views that give them reasons and motivations to act in certain ways and that might affect their professional practice. After consultation, Jane’s doctor refuses to provide the abortion and Joanna’s doctor offers the service.

#### Sterilization

Emma and Cassandra are both twenty-five years old. Neither currently has children and they have both decided against having children in the future. They both approach their doctors to ask for sterilization. Emma’s doctor is a devout Roman Catholic, who is personally against contraception of any kind. Cassandra’s doctor on the other hand is a committed environmentalist who feels strongly that, as a species, we are having too many children for a sustainable future for the planet. After a consultation, Emma’s doctor refuses the sterilization, and Cassandra’s doctor agrees to the sterilization. We do not know whether the doctors’ values affected their consultation or the outcome.

In each of these examples the professionals have personal motives, values, perhaps goals that are likely to—though in practice may not—affect their professional conduct. Is this a problem? Should these doctors be prevented from acting in accordance with their personal motives or beliefs? Are there any significant differences among the cases above, and therefore should they be treated as ethically distinct scenarios that need to be managed differently? These are the questions we will address in this paper.

## Financial Conflict of Interest and Non-Financial Conflict of Interest

According to current guidelines regulating healthcare practice and biomedical research and scholarly debate on this issue, in the scenarios described above, the only problematic interactions between the doctors’ personal lives and their patient care arise for John’s doctor in the antibiotics scenario and perhaps Mark’s doctor in the vaccination scenario (see, e.g., Rodwin 1993; Topol and Blumenthal 2005; Brennan et al. 2006; Brody 2011; Rodwin 2011; Rodwin 2012; Stamatakis et al. 2013; Kelly 2016). This is because they involve financial interests, which are typically taken to generate either the *only* or the *most problematic* type of conflict of interest (COI) in healthcare. The U.S. National Institutes of Health rules for dealing with conflict of interest in medical research and the U.K. General Medical Council guidelines on the subject only address financial conflict of interest (FCOI) (Department of Health and Human Services 2011; GMC 2013). The NHS definition of “interests” in its document regulating COI management, while including non-financial interests, understands them merely in terms of material gains or of some form of personal benefit, such as professional reputation (NHS England 2017). Where the possibility of non-financial conflict of interest is acknowledged (e.g., Institute of Medicine 2009), the tendency is to think that non-financial interests affecting professional healthcare practice *should not* (Bero and Grundy 2016) or *cannot* (Institute of Medicine 2009) be regulated. In the scholarly debate, only recently have some started to include non-financial interests among the possible sources of COI that might need to be prevented, constrained, or somehow regulated in the same way as financial interests, both in biomedical research (Saver 2012) and in healthcare (Wiersma et al. 2018a and 2018b; Smith and Blazeby 2018).

It is noteworthy that some of the interests that we have mentioned in the above scenarios, namely those having to do with certain moral beliefs, are not only considered permissible but are often supported in academic and professional contexts, including through legislation in the form of “conscientious objection” provision. Nonetheless, as some of the scenarios above suggest, personal moral or religious beliefs can affect one’s professional practice to the same extent as, if not to a greater extent than, financial interests.

In this paper, we will argue that ethical issues arising from conflicts of interest in healthcare are not limited to financial interests, and that the same ethical and regulatory perspective currently adopted with regard to FCOI should be extended to non-financial conflict of interest (NFCOI). Not all the situations in the scenarios above constitute a conflict of interest, and not all COIs in healthcare give rise to an ethically impermissible outcome. However, we argue that the issue of whether a COI is financial or non-financial in nature is not the criterion by which to determine whether a certain interest generates a conflict of interest or whether that conflict led to unethical behaviour. NFCOIs have the potential to lead to ethically impermissible or ethically problematic behaviours just as FCOIs can, and they should be managed to avoid this outcome to the same degree as FCOIs, if doing so is feasible. In order to argue for this claim, we need first to clearly define what is meant by “conflict of interest,” which requires some preliminary analysis of the concepts of “interest” and of “conflict.” We will turn to this task in the next section.

## Interests, Conflicts, and Conflicts of Interest

### Interests

From a philosophical point of view, an interest can be defined in terms of having a stake in something, or, as Joel Feinberg puts it,

[o]ne’s interests (…) taken as a miscellaneous collection, consist of all those things in which one has a stake, whereas one’s interest in the singular, one’s personal interest or self-interest, consists in the harmonious advancement of all one’s interests in the plural. (Feinberg 1987, 34)

An alternative but equivalent characterization, still inspired by Feinberg, is the one according to which “to have an interest in something is to have a stake in it, and to have a stake in *X* is to stand to lose or gain depending on what happens to *X*” (Weale 1998).

The interests in question when we discuss COI in healthcare are specific interests that can conflict with either other individuals’ interests (most notably, patients’ interests) or with specific professional obligations. The reference to professional obligations is important. An understanding of COI in healthcare that focuses only on the conflict between a physician’s personal interests and the interests of patients is too narrow to capture the ethically relevant aspects of conflicts of interest in healthcare. Brody’s definition of conflict of interest, based on Erde’s influential analysis (1996), seems to imply this narrow understanding when he writes that an interest that gives rise to a COI in healthcare is one that “would tempt a person of normal human psychology to neglect the patient’s/public’s interests in favor of the physician’s (or third party’s)” (Brody 2011, 24).

Sometimes, however, physicians have personal interests that do not conflict with individual patients’ interests or the public interest but that do conflict with their obligations to the profession. One example is an obligation to act according to a certain standard of fairness, for example in the distribution of the burdens required by certain professional roles amongst the workforce. These professional obligations can be taken to be “interests,” or “professional interests,” according to a different, and broader, definition of “interest,” such as the one provided by the Royal Australasian College of Physicians, according to which an interest is to be understood as “a value, goal or obligation associated with a social relationship or practice” (The Royal Australasian College of Physicians 2018, 7). The word “associated” here can be understood either in a descriptive sense (as a matter of fact, certain relationships or practices do generate interests for the physicians, such as receiving gifts from pharmaceutical companies in exchange for promoting certain drugs) or in a normative sense (certain practices, such as the healthcare profession, *should* generate interests for professionals to fulfil certain professional requirements). In other words, the normative sense suggests there is something wrong when one has an interest in practising within a profession but does not have an interest in fulfilling the requirements of that profession. In fact, in most professions, failing to fulfil professional obligations is a reason for dismissing a person from their role.

We have thus distinguished what we might call “personal interests” from what we might call “professional interests.” A general definition provided by Lipworth et al. (2019) is broad enough to include both types of interests; as they define them, interests are “people’s concerns for themselves or perceived duties to others that are relevant to the social role or roles they assume.” However, it is useful to keep the two kinds of interest distinct in order to more easily see how they can conflict with each other in healthcare. The scenarios described at the beginning contain clear examples.

Below*,* we will return to the issue of how professional obligations should be defined on the basis of the “four principles” of biomedical ethics: respect for autonomy, beneficence, non-maleficence, and justice (Beauchamp and Childress 2012).

### Self-Interest

When a conflict does arise, one factor which differentiates the FCOIs and NFCOIs might be the question of *self-interest*. A doctor with an FCOI is clearly party to a *self-interested* benefit by prescribing, for example, unnecessary antibiotics from a company the doctor holds shares in. On the other hand, an NFCOI is harder to connect to self-interest.

However, there may be directly analogous self-interested benefits arising from non-financial interests also. Status and recognition are one example, as are doctors’ personal moral or religious views—what we might call “moral interests.” Importantly, moral interests also have implications for one’s status and standing to others and oneself. Someone’s conscientious objection to abortion might help them gain social recognition among colleagues or superiors who share the underlying moral beliefs. Thus, moral interests may be a more potent source of conflict than money, both because of a professional’s interest in preserving their own moral integrity and because of their interest in gaining some form of recognition within a certain group (say, colleagues or superiors with the same moral convictions, their religious community, and so on).

However, even if we assume that the doctor does not receive a *self-interested* benefit in this way, or even if the doctor experiences a social or professional cost for their behaviour, the problem that a COI poses is not that the doctor receives a self-interested benefit *in itself*. After all, we tend to pay doctors well for their work, and it would be perfectly acceptable for a doctor to choose one arm of the profession over another for its better rate of remuneration. A problem arises if and when, due to the COI, the patient’s care was affected by the interest, or was seen to be affected.

Consider a COI in the legal profession. A solicitor cannot act for both parties in a house purchase. This is not to avoid *self-interested* behaviour. It is because a solicitor has a duty to put each client’s interests first. It is not possible to commit to this for both clients. Each time she meets a client, there is the potential that her consultation will be affected by her other client’s interests.

### Conflicted Care

Of course, in professional settings, and in the healthcare profession in particular, not all personal interests generate a *conflict* of interest. For instance, an interest in winning the prize for best healthcare practitioner of the year might motivate a professional to fulfil their professional obligations as best as they can. The financial interest and the interest in gaining status and recognition in this case align with, and indeed promote, the best practice fulfilment of professional obligations.

A conflict arises if a consideration outside of best practice in the individual circumstances is, or could be seen to be, affecting a professional judgement. These become ethically unacceptable when they are allowed to in fact have an influence over professional judgement so that the patient is given treatment or care which deviates from the best practice in their individual circumstances.

The case of Mary’s doctor in the antibiotics scenario at the beginning is an example of overlapping personal interest and professional obligation: as a doctor acting on the best evidence available, she should not provide unnecessary antibiotics. Her behaviour is not unethical, even if she was firmer in her resolve not to provide unnecessary antibiotics because of her personal history. It merely bolstered her adherence to the best care for her patient. If, however, in a separate case, she refused a patient with a bacterial infection the appropriate antibiotics, for whom antibiotics were medically indicated even accounting for the public health cost in terms of ABR, because her mother’s death led her to believe that not prescribing them would save other patients, she would indeed be acting unethically. Financial COIs may also be benign in practice. In the vaccination scenario, although Mark’s doctor has an FCOI, it does not necessarily affect his ability to provide the best-practice care, which is, with appropriate informed consent, to provide the very vaccine he has a financial interest in.

Non-financial sources of conflict are often overlooked as COIs (Wiersma et al. 2018a and 2018b). Yet the religious doctor in the abortion scenario who refuses an abortion has a personal belief that prohibits her from providing the medical care that her patient is entitled to.

The important factor in judging whether a doctor has behaved unethically in relation to a COI is not the type of interest the doctor held, or even whether the doctor was self-interested, but whether the medical advice or treatment that was in fact provided was in line with what should have been provided for the patient according to the principles of medical ethics. Mary (antibiotics) and Mark (vaccines) did receive the best treatment. The COI might nevertheless still need to be somehow managed, so that the patient may assess the advice appropriately and be assured that they did indeed receive professional judgement in line with best practice. This includes providing the appropriate support to a patient in the course of decision-making, as well as the appropriateness of the final decision. Importantly, doctors should engage in dialogue with patients articulating their reasons for recommending an intervention (Savulescu 1995). In the sterilization case, the environmentally concerned doctor appears to act in line with the patient’s autonomous choice. However, we do not know the basis of the patients’ decisions or their understanding of the procedure and risks and so on. It may be that only the religious doctor explained them dispassionately to her patient, and in fact offered the best medical practice, or that both did, and the patients autonomously came to different decisions based on the same facts and values as they applied them to their own lives.

In the case of Joanna (abortion) and John (antibiotics), the patients did not receive the best care. Failure to provide the best treatment could be caused by a number of factors (poor training, human error, social factors such as pressure to prescribe in the antibiotics case). However, if it does arise from a conflict, the doctor should be held accountable by specific regulation.

COIs should be managed to ensure that all patients receive the best treatment to which they are legally entitled, regardless of any COIs, financial or non-financial.

### Freedom

A potential difference between FCOIs and NFCOIs concerns the scope of our personal freedom. Here, the ethical issues that COI raises are different when we consider society in general and specific professional contexts.

To borrow again from Feinberg,

… not all invasions of interest are wrongs, since some actions invade another’s interests excusably or justifiably, or invade interests that the other has no right to have respected. The interests of different persons are constantly and unavoidably in conflict, so that any legal system determined to “minimize harm” must incorporate judgments of the comparative importance of interests of different kinds so that it can pronounce “unjustified” the invasion of one person’s interest of high priority done to protect another person’s interest of low priority. (Feinberg 1987, 35)

Such judgements of comparative importance can be difficult to make when interests are managed through poorly defined principles such as “freedom of conscience.” For instance, most of us agree that individual freedom should not extend so far as to significantly harm other people in the name of one’s religion or one’s political views. But what does this mean in practice? In some cases, there can be little doubt that the legitimate boundaries of, say, religious or political interests and freedom have been crossed. For instance, killing in the name of one’s religion or one’s political views is not acceptable, no matter how strong one’s religious or political interests are. But we may legitimately be required to endure a level of discomfort, or even harm, to accommodate these interests—for instance, when a political rally paralyses a city. It can be difficult to assess the legitimate boundaries of these freedoms. For instance, does religious freedom warrant pro-life campaigners holding rallies outside clinics where abortions are performed? There may be reasonable disagreement over the balance between the value of religious freedom and the disvalue of psychological harm to women. The same need to balance competing interests applies to financial interests. An individual’s right to private property and to make a profit often comes into conflict with the interests of others, and it can be difficult to assess the extent to which these interests are invaded excusably or justifiably, to use Feinberg’s terminology.

The boundaries between different rights and freedoms are an issue that society continues to struggle with. However, specific sectors within society have their own rules that provide clear guidance about how the vague principles mentioned above—e.g. freedom of conscience, right to private property, and so on—should be applied. In particular, properly regulated and recognized professional settings such as the healthcare profession have specific professional and ethical requirements that apply to those who freely choose to enter that profession.

### Professional and Ethical Requirements in Healthcare

In the case of healthcare, professional requirements relate to the best practice distribution of the legally available treatments in order to achieve the best medical outcome(s). The precise ethical guidelines may vary from jurisdiction to jurisdiction, but Western medicine and healthcare is based on the widely accepted principles of patient autonomy, beneficence, non-maleficence, and justice in allocation of healthcare resources (Beauchamp and Childress 2012). The “principlist” approach has its own shortcomings and critics, but it has two elements to recommend it.

First, none of the four principles we have just mentioned is itself questioned in contemporary secular medical ethics, at least in Western societies. The critical discussion focuses on whether principlism can work *as a system* and whether it is a comprehensive approach. For example, one could ask whether additional principles need to be included (e.g., solidarity, dignity, integrity (Rendtorff 2002)) and whether there should be an external criterion for ranking these principles when they conflict with each other (see, e.g., Huxtable 2013; Walker 2009; Callahan 2003). But respect for patient autonomy, commitment to fair treatment, and the requirement to act in a patient’s best interests (to the extent that this is medically possible) are widely accepted professional expectations. In addition, there are general professional standards that apply to any sector, including upholding a minimum work ethic, fulfilling the requirements of the role (that is, basically, what one receives a salary for), and respecting and treating one’s colleagues fairly. Unfair treatment of colleagues may include overburdening them with a workload above or outside of their role.

Second, even if one thinks such principles are questionable (either individually or as a system), as a matter of fact they are the principles informing professional standards in most healthcare systems, at least in Western countries.

Now, not every legally available medical intervention meets the above standards of medical ethics. One example is capital punishment, which is legal in certain contexts but is not consistent with medical ethical principles of autonomy, beneficence, and non-maleficence. Personal moral views against capital punishment in these cases are therefore not in conflict with a doctor’s duty to his or her patient. Of course, we are not interested here in discussing capital punishment from an ethical-legal point of view. Our point is simply that what constitutes a conflict of interest is determined by the ethical standards of a profession and not necessarily by what the professionals are legally allowed to do.

Some believe that abortion falls into the same category as capital punishment: even if it is legally available, it is against the principles of medical ethics. But medical ethics is not relativist. It does not depend on what one thinks or earnestly believes is right or wrong (Savulescu and Schuklenk 2017). Abortion is taken to be consistent with the accepted ethical standards of the healthcare profession, and rightly so: it can be in a woman’s best interest and it does not go against any sufficiently morally significant interest of the foetus, including the interest in living, given certain plausible philosophical assumptions. Arguing for this claim here is beyond the scope of this paper, of course. Our point is simply about the proper scope of healthcare practitioners’ personal ethical views in the exercise of their profession: we should not be relativist and give those the same ethical weight as the accepted ethical standards of the profession. We do acknowledge that both ethical and professional medical standards in their current form may be, in fact, mistaken. There are many examples in history of both medical and ethical best practice that has subsequently been shown to be wrong. However, the space to question and try to reform the standards of one’s profession exists in liberal democracies and it is right that doctors and other stakeholders have a voice in these issues. But an individual doctor should not take it upon themselves to unilaterally impose their own beliefs on individual patients against currently accepted principles of professional ethics. This would be a form of relativism. Also, among other things, doing this would violate a principle of justice, as patients receive medical care based solely on their doctor’s own views.^1^

As a comparison, consider a doctor who takes an interest in homeopathy. Disheartened by evidence of widespread harm caused by pharmaceuticals such as “pharmageddon,” and some of his own patients suffering side-effects, the doctor comes to earnestly believe that homeopathy is a better option for all patients than mainstream medicine. He prescribes it instead of the pharmaceuticals that have been approved for these conditions. This would be professional malpractice in most contexts and by most medical standards (with exceptions, though; for instance, it is still quite common in a country like Germany for doctors to prescribe homeopathy).

### Conflict of Interest in Healthcare

Now, having a personal interest that conflicts with professional obligations is not per se ethically impermissible. Indeed, every one of us is likely to have such conflicts of interest— at least in the sense in which we have defined these terms here—regardless of their profession. There are often aspects of our jobs that we would be better off—financially or in some other respect—not doing or that conflict with some of our moral beliefs or some other interests we have. The ethical problem arises not when we *have* such interests but when we *prioritize* such interests in a way that influences or even prevents us from fulfilling our professional obligations, for example, delivering ethical medicine.

We can avoid existing conflicts becoming “morally culpable” by managing them in a way that doesn’t lead to violations of professional obligations (Lipworth et al. 2019). Receiving financial gifts from pharmaceutical companies is not wrong per se. It is only wrong when these interests influence practice in a way that affects the fulfilment of professional obligations. A second issue arises that it can be difficult to establish when such influence plays a role in clinical decision-making. In the scenarios described above, whether the conflict of interest has led the doctor to act unethically is something we do not know and that would be very difficult, if not impossible, to establish. For example, in the antibiotics scenario, we do not know whether gifts from pharmaceutical companies influenced the first doctor’s decision to prescribe unnecessary antibiotics. Clinically unjustified antibiotic prescription is a widespread habit, and financial conflicts of interest are only one possible factor (other factors include patient pressure to prescribe).

Thus, even if COIs are not in themselves unethical, they present a risk of ethically impermissible behaviour *if and when* they translate into unprofessional behaviour. The mere *appearance of* a COI influencing a clinical decision may also undermine overall trust in the individual decision or in the profession as a whole. There is therefore a reason to regulate COIs that *have the potential to* translate into unprofessional behaviours, regardless of whether they *in fact do*.

While this might be fairly easy to determine in most cases of FCOIs, in the case of NFCOIs it may be more difficult to point to a consistent bias that affects professional conduct. Some take this to be a reason not to regulate NFCOIs (Bero and Grundy 2016). A regulation needs to be not only ethically justified but also feasible and sufficiently easy to implement.

While the feasibility of regulation might provide a reason to treat FCOIs and NFCOIs differently, nothing we have said so far suggests that only financial interests can generate COI or that only FCOI can be ethically problematic or impermissible. And indeed, the definitions of COI in healthcare commonly provided in the literature, even when such definitions are meant to refer only to FCOI, do seem to be applicable to both types of interests. For instance, Erde’s and Brody’s definition of COI reported above applies equally well to the case of financial and to the case of non-financial interests, even if it is meant to apply only to FCOI. According to Brennan and colleagues, “[c]onflicts of interest occur when physicians have motives or are in situations for which reasonable observers could conclude that the moral requirements of the physician’s roles are or will be compromised” (2015, 430). This definition equally applies to financial and non-financial interests and closely resembles the one provided by NHS England, with the only difference that the NHS includes both actual and potential conflicts (NHS 2017). The U.S. Institute of Medicine says that “a conflict of interest is a set of circumstances that creates a risk that professional judgment or actions regarding a primary interest will be unduly influenced by a secondary interest” (IOM 2009, 46). Once again, such definition applies equally well to financial and non-financial interests.

Thus, non-financial interests can be in conflict with professional obligations and NFCOI can be as ethically problematic as, if not more ethically problematic than, FCOI (Lipworth et al. 2019). A financial interest in prescribing the HPV vaccine to a male child does not become ethically problematic if the doctor’s treatment aligns with best medical practice. A non-financial interest such as a commitment to a religious or ethical view that discourages a doctor from providing an abortion, sterilization, or a vaccination is ethically problematic if it hinders the provision of best medical care.

Now, we have mentioned above that, even if in principle FCOI and NFCOI should be treated in the same way, there will *often* be practical reasons for managing them differently—or even for managing the former and not the latter (Bero and Grundy 2016). Often, but not always. As we will argue, the ethical equivalence of FCOIs and NFCOIs has implications for how at least one type of NFCOI should be regulated, namely the conflict that results in conscientious objection in healthcare. We claim that conscientious objection in healthcare should be conceptualized as the expression of a type of COI, and more precisely, ethically impermissible COI. Also, we claim that management of the COI that is currently covered by conscientious objection in healthcare would be feasible. We will suggest that conceptualizing conscientious objection in healthcare as a form of ethically impermissible management of a conflict of interest lends support to a position against a right to conscientious objection in healthcare.

### Conscientious Objection as a Conflict of Interest

Conscientious objection in healthcare is the refusal by healthcare personnel to perform or take part in certain professional activities because they conflict with their own personal moral or religious beliefs (see, e.g., Savulescu 2006; Card 2007; Sulmasy 2008; Brock 2008; Wicclair 2011; Savulescu and Schuklenk 2016; Giubilini 2017). Of course, sometimes it is difficult to disentangle clinical judgements from value judgements based on personal beliefs. The line between the two can be blurred. Here, however, we are only discussing conscientious objection understood as the situation in which a professional’s personal values are allowed to prevail over clinical judgements.

Conscientious objection procedures are in place in many jurisdictions, allowing healthcare practitioners to abstain from certain medical services, such as abortion, end of life decision-making, and providing or prescribing contraceptives.^2^ Conscientious objection can take different forms. For example, some policies require referral to a non-objecting colleague and some do not. Some require patients to be informed of all available options and some do not. In all cases, conscientious objection clauses allow the healthcare professional to provide the patient with a medical service that is not led by the standard of best practice for that patient according to current medical and ethical guidance but by the practitioner’s own non-financial interests. This is the precise situation that COI policies should be designed to prevent. We will argue that a more appropriate policy for NFCOIs, and particularly those that find expression in conscientious objection, would be to mirror FCOI policies as far as is feasible.

#### Interests

According to the notion of “interest” as we have analysed it above, the moral or religious views that motivate conscientious objection do constitute interests in the same way as the financial and non-financial interests that are usually the focus of discussion around COI in healthcare. They all trigger motivations and goals that are relevant to and can affect one’s professional practice. Indeed, often religion represents a stronger commitment to a certain goal than money. Some doctors who are opposed to abortion would go so far as to put the life of a pregnant woman at risk in order to avoid committing what they perceive to be murder. Some would refuse to refer a pregnant woman seeking an abortion to a non-objecting doctor or to inform her that abortion is an option, in order to avoid being complicit in wrongdoing (Minerva 2017). This is plausibly attributable to both or either a sense of self-respect that comes from protecting one’s own moral integrity (Wicclair 2000; Sulmasy 2008) and/or the desire to protect one’s reputation and standing in the eyes of one’s own group (Dawson et al. 2017; Keogh et al. 2019). If we consider what might amount to a comparable FCOI, it is plausible to suppose that it would take a very large sum of money to be considered sufficient incentive for a physician to act against the best interest of a patient whose life is at stake.

#### Conflicts

Interests arising from conscience can conflict with professional obligations. We have suggested above how these types of interests could conflict with a patient’s best interest, for instance the best interest of a woman who needs and autonomously requests an abortion. But importantly, refusing to act in the best interest of their patients is not the only way a healthcare professional can fail to fulfil their professional obligations. For instance, like all other forms of interests that affect one’s professional practice, conscientious objection can violate basic requirements of fairness. Where women have a right to safe and legal abortion, and considering that only certified healthcare professionals are authorized to perform them, the body of healthcare professionals has a collective moral obligation to guarantee that women can access the service. The obligation is collective in the sense that, given certain individual rights to obtain certain services and given the monopoly that a professional body has over that service provision, it is the profession with that monopoly that has the responsibility to fulfil those rights. No one else can. The question is, of course, what it means, in terms of attribution of responsibility to individual agents, to say that a profession has a certain collective responsibility.

The burden of the collective obligation ought to be shared fairly among individual members of the collective, at least if we accept the principle that fair equality of rights and duties should be applied in properly regulated professional settings. If a certain service is part of a profession, someone who chooses that profession has no legitimate claim to be exempted, given that after their conscientious objection has been granted their work conditions (e.g., their salary) would normally remain the same as those of someone who does provide the service in question. So, these two individuals would have equal professional rights (e.g., to a certain salary) but unequal professional burdens. Nor can the unfairness be rectified by creating incentives for those who do agree to provide the service above what is due to them anyway; again, it would be unfair to use resources to create incentives for doing something that in any case falls within the scope of the role, unless such resources are created by disincentivizing (e.g., through salary cuts) conscientious objection. But those who defend a right to conscientious objection typically do not accept penalties for failing to provide services on conscience grounds (for a discussion, see LaFollette 2017).

The point we have just made applies especially when professionals with and without conscientious objection receive the same salary from a public healthcare system. However, the same consideration can be extended to private healthcare provision to the extent that healthcare professionals have the monopoly over the provision of certain services that citizens have a right to receive: if the professional body has an obligation to guarantee the service, the fact that professionals are employed in the private or public sector does not make a difference to what counts as fair distribution of the burden. Private healthcare providers are still healthcare providers, that is, holders of a monopoly over something citizens have a right to receive.

A common solution in conscientious objection clauses is to require referral. However, referring a patient to a doctor who would be willing to provide the service does not fulfil the fairness requirement because it would generate an extra burden for this other professional without placing any commensurable burden (or indeed any burden at all) on the objecting doctor. It also places additional burden on the patients, particularly patients who are poorer (even in public health service settings, as appointments may represent time away from paid work) and/or rurally based.

It is important to emphasize once again that here we are only talking of conflicts generated by professionals’ *personal* moral views not conflicts between any ethical values in general. For instance, allocation of scarce healthcare resources is an ethical decision that involves conflicts of values, such as patient autonomy and fairness. However, the values involved should not be the personal moral beliefs of any individual healthcare practitioner. They should be determined and weighed on the basis of professional and legal standards (Savulescu 2006).

## Is Conscientious Objection Ethically Equivalent to Financial Conflict of Interest?

The implication of the analogy between NFCOI and FCOI is pretty straightforward: allowing a NFCOI to interfere with the fulfilment of one’s professional obligations is ethically equivalent to allowing a FCOI to interfere with the fulfilment of one’s professional obligations*, other things being equal*. Thus, the equivalence suggests that NFCOI should be managed in the same way as FCOIs to ensure that they do not impact patient care. Conscientious objection policies, in contrast, work to support the place of the non-financial interest in the provision of healthcare.

Now, of course the analogy would support equal treatment of NFCOI only if other things were equal. If there were significant differences between FCOI and NFCOI in practice that made equal treatment ethically not permissible *all things considered* or practically unfeasible, as we mentioned above, then that would be a reason to treat them differently.

We can identify three significant differences between FCOI and NFCOI that might be taken to justify different treatment, and that might make a policy of disclosure of NFCOI unfeasible. However, we will show that although they might apply to many forms of NFCOI, they do not apply in the specific case of conscientious objection.

First, generally, non-financial interests are very difficult to detect, unlike most financial interests (Institute of Medicine [IOM] 2009). Often, the professional themselves might be unaware of these interests, as they might simply be the result of implicit biases (e.g., sexist attitudes or status quo bias). Financial interests are more objective and easier to detect. Also, even where NFCOI can be detected, it is difficult to show resulting consistent bias that would render the NFCOI ethically impermissible (Bero and Grundy 2016). Hence, managing FCOI seems feasible in a way in which managing NFCOI often does not.

Second, one might argue that it is unreasonable or ethically unacceptable to require disclosure of certain non-financial interests, for instance because disclosure would violate privacy rights with regard to personal information and could lead to unfair discrimination. For instance, requiring someone to disclose religious or ethical views or relevant personal history that might affect their professional conduct (such as the doctor in the antibiotics scenario whose mother died of a resistant infection) might force individuals to disclose personal sensitive information, which is more problematic than requiring them to disclose their sources of financial profit.

Third, it might be discriminatory to manage certain NFCOIs in the same way as we manage FCOI, since personal values and beliefs should normally not, by themselves, prevent individuals from enjoying fair equality of opportunities, including the opportunity to pursue certain careers. There is no reason to assume that a doctor who self-identifies, for instance, as a Catholic or an environmentalist will not be a good doctor. A doctor who meets professional standards should not be discriminated against on the basis of religion or other values. In contrast, prohibiting FCOI would not give rise to unfair discrimination in most cases. Although in general people are allowed to make a profit in any way that is legal, and although in principle one can have FCOIs that are not ethically impermissible—that is, that do not actually interfere with the fulfilment of one’s professional obligations—it can be reasonable to require abandoning certain conflicting financial interests. A reason for this claim is the one provided by Bero and colleagues, according to whom

… an investigator can divest themselves from shares in the company that commercializes their research product, whereas they cannot possibly separate themselves from their disciplinary training. Similarly, if the only solution for a particular type of interest is recusal because the interest cannot be eliminated, this is not a conflict of interest but rather part of the researcher’s professional role or personal identity. (3)

They think this is a rule of thumb to distinguish “conflicts of interest from interests more broadly” (3). Bero and colleagues are right to suggest that financial and non-financial interests are different in that only the latter are constitutive of one’s “personal identity,” in the sense of one’s understanding of who or what kind of person one is. For this reason, it is more ethically problematic to prevent people with certain non-financial interests from entering a profession than it is to prevent people with certain financial interests from entering the same profession. The degree of unfairness, particularly in terms of unfair discrimination, in denying access where there is NFCOI seems greater than the degree of unfairness where there is FCOI.

These differences might justify treating FCOI and NFCOI differently in most cases. Thus, even if in principle conflicts of interest are ethically equivalent regardless of whether they are financial or non-financial in nature, there might be other good ethical reasons (such as unfair discrimination and privacy rights) and practical reasons (such as difficulties in detecting sources of conflicts) to only regulate FCOIs. However, what are the implications of these differences?

The implication is that ethically impermissible FCOIs and NFCOIs should be treated differently *only* when treating them in the same way would come at the cost of disregarding these other values. Admittedly, that cost may arise in many, perhaps even most, cases. For instance, requiring doctors to disclose their personal history might violate a doctor’s right to privacy. Prohibiting a Catholic from pursuing a career in healthcare because of their religious *beliefs* is a form of unfair discrimination. However, it does not follow from those disanalogies that conscientious objection in healthcare should be allowed as it normally is today, because allowing conscientious objection would not solve any of the three problems we have described. Let us consider them in order.

The first disanalogy does not represent a reason for allowing conscientious objection in healthcare. The difficulty in detecting non-financial interests and in identifying consistent bias weighs in favour of constraining only FCOI because it would be difficult to identify and quantify the influence of non-financial interests. It might not even be clear what types of non-financial interest we would need to target. But when it *is* clear that certain well identified non-financial interests are affecting professional conduct, this problem does not arise. Conscientious objection presupposes precisely that the non-financial interest be disclosed and, therefore, detected. It is because it is known that a professional has certain moral beliefs that this professional is allowed to object to providing a certain service. Allowing conscientious objection means precisely allowing certain well-identified personal religious or moral views to affect one’s professional practice. This is not the standard approach to FCOI. If the analogy with FCOI holds, then this specific disanalogy between NFCOI and FCOI does not seem to apply to the case of conscientious objection and therefore does not constitute a reason for regulating conscientious objection differently from ethically problematic FCOI.

A similar consideration can be made about the second disanalogy. If disclosure is required, of course, as Wiersma and colleagues point out, non-financial interests, some of which may be highly personal, must be handled with discretion to avoid needlessly intruding into people’s privacy or placing them at risk of discrimination” (Wiersma et al. 2018b,K1240). However, this consideration does not seem to apply to the case of conscientious objection: allowing conscientious objection presupposes precisely the full disclosure of personal information which might be thought of as protected by privacy rights, such as religious beliefs. Since the ground for conscientious objection lies in a principle of “freedom of conscience” and “freedom of religion,” disclosure of one’s conscientious or religious beliefs is a necessary condition for granting conscientious objection because it ensures that those are the principles we are appealing to and that we are not merely protecting other kinds of preferences (such as preferring to avoid unpleasant procedures like abortion). Forced disclosure of personal sensitive beliefs, such as religious beliefs, might legitimately be prohibited in order to avoid unfair discrimination, but in the case of conscientious objection the disclosure does not lead to unfair discrimination. Quite the opposite: it benefits those who choose to disclose certain religious or moral beliefs (such as those that explain their refusal to perform, say, abortion) because, through conscience exemptions, they are granted what is arguably a privilege (since, as we said above, they would enjoy equal rights but unequal burdens compared to their colleagues without conscientious objection).

The third difference is that excluding people with certain ethical or religious beliefs from one’s profession is discriminatory. This might seem to be the one that weighs most heavily in favour of allowing conscientious objection while constraining ethically impermissible FCOI. However, this is not the case. It is unfair to exclude people from certain professions on the basis of their religious or ethical beliefs *alone*. It is not unfair to exclude them from certain professions on the basis of the behaviour that causes them to fail to perform their job (in this case delivering medicine consistent with ethical principles) that results from certain beliefs. Freedom of conscience, thought, and religion—which is protected by the U.N. Universal Declaration of Human Rights—is often taken to be coextensive with the freedom *to act* according to one’s conscience, thought, and religion. This is mistaken. One should not be free to act in these ways when it harms others or where it causes a doctor to fail her patient. Circumstances matter, rights are not absolute, and therefore different circumstances might constrain individual rights. In particular, freely deciding to pursue a certain profession inevitably puts legitimate constraints on other freedoms and most notably the freedom to act in a certain way.

Beliefs and behaviours often go hand in hand in liberal societies, and they should be allowed to go hand in hand to the greatest extent possible, but they are not the same thing. Prohibiting conscientious objection in healthcare would only constrain freedom of conscience, thought, and religion to a certain, permissible degree, because it would only prevent one aspect of such freedoms that could be ethically impermissible, that is, freedom of action. In professional settings, what is ethically permissible or impermissible is the behaviour, not the belief. Appealing to freedom of conscience and religion to defend conscientious objection in healthcare is a form of “conscience absolutism” which mistakenly presupposes that certain rights are absolute.

### Solutions

We have argued so far that conscientious objection in healthcare should be regulated in the same way as ethically problematic FCOI. But how should they all be regulated, exactly?

The solutions usually offered to address conflict of interest in medicine (and in biomedical research)—which have typically been developed solely with FCOI in mind—are generally meant to preserve professionalism and trust between healthcare providers and patients (Kelly 2011; Brody 2011). There are basically two kinds of solutions. One is a disclosure requirement. The other is prohibition of any conflicting financial interest (Brennan et al. 2005), at least of significant size in the case of gifts (NHS 2017).

The mere disclosure requirement is the policy approach preferred by most medical organizations (Brennan et al. 2005). The requirement is that doctors and researchers disclose financial interests that may reasonably be expected to come into conflict with their professional obligations from time to time, even if they do not come into conflict in all cases (Topol and Blumenthal 2005). For instance, receiving sufficiently large financial gifts or other forms of material incentives (say, hospitality and conference invitations) from pharmaceutical companies creates a COI in the antibiotic scenario but not in the vaccination scenario, since administering vaccines is a professional obligation while prescribing unnecessary antibiotics violates a professional obligation. One reason for requiring disclosure of such interests is that disclosure would make it more difficult for such interests to result in ethically impermissible professional behaviour, as it would make it easier for patients and/or institutions to detect or foresee a deviation from professional standards. However, some are sceptical that disclosure would be sufficient to counteract the problem of COI. According to Brennan and colleagues, for instance, “physicians differ in what they consider to be a conflict, which makes the disclosure of conflicts incomplete” (2015, 431). Some people think that in many cases non-financial interests such as religious views could be managed through disclosure in the same way as financial interests are (Wiersma et al. 2018a). Whatever the merits or demerits of the disclosure requirement in the case of FCOI—something we are not taking a stand on—it does not represent a solution to the problem of conscientious objection in healthcare. Disclosure is meant to ensure that ethically impermissible COI—that is, behaviours informed by the conflicting interests—do not occur, and to reassure patients that there is a transparency. But as said above, the institution of conscientious objection in healthcare goes in the exact opposite direction, as disclosure is used to ensure that ethically impermissible NFCOI *does* occur.

Prohibition of all financial gifts is a more extreme solution and one that some scholars (Brennan et al. 2005; Rodwin 2011, 23) and medical institutions (IOM 2009) have advocated. For instance, according to Brennan and colleagues,

… because gifts of even minimal value carry influence and because disclosure is an inadequate safeguard […] [a]ll gifts (zero dollar limit), free meals, payment for time for travel to or time at meetings, and payment for participation in online CME from drug and medical device companies to physicians should be prohibited. (Brennan et al. 2005, 431)

However, this might not be ethically justified as a matter of principle, since as we have seen, not all financial interests generate FCOI nor do all FCOIs lead to improper decision-making. But there might be prudential and feasibility reasons for implementing such a policy, since it might be too difficult to determine when and to what extent any single financial interest does result in ethically impermissible behaviour. Current policies usually seek to manage FCOIs according to the degree to which they might affect, or be seen to affect, professional judgement. A range of measures are available to manage such FCOIs, ranging from no action, to limiting the types or amounts of gifts that may be accepted, to removal of the professional from the role altogether.

Even if outright prohibition of FCOIs is too extreme a measure and ultimately not justified, the analogy between FCOI and NFCOI still supports banning conscientious objection. If we thought that prohibiting any FCOI is too extreme, it would not be because ethically impermissible FCOIs are, all things considered, deemed acceptable but simply because we should make a bigger effort to detect which FCOIs are ethically permissible and which are not. Allowing conscientious objection, on the other hand, implies precisely that an NFCOI may affect professional judgement and patient care. If the analogy between FCOI and NFCOI holds, and we should manage NFCOIs to ensure that they do not affect professional judgement, then conscientious objection, which manages NFCOIs precisely by providing a route for non-medical factors (such as the religion of the physician) to influence patient care, should not be allowed.

A Few Practical Guidelines

1. Where a doctor’s personal values conflict with the accepted professional and ethical standards, they should be disclosed.
2. The doctor should engage the patient in dialogue outlining reasons for the recommended course of action in terms of medical ethical principles, such as the four principles of biomedical ethics.
3. Personal values should not be allowed to compromise patient care: healthcare professionals should provide the agreed service if it is medically indicated and in line with professional standards.
4. Where this is not possible, the priority should be the provision of patient care in line with professional standards. This might mean a range of measures, including a change of roles to a role where the COI will align with the provision of patient care.

### Conclusion

If we frame conscientious objection as the expression of a conflict of interest in healthcare, then it is apparent that, at the moment, conscientious objection is treated and managed differently from the way other conflicts of interest are treated and for no apparent good reason. Allowing conscientious objection to certain practices means not only acknowledging that a conflict of interest exists (because we are acknowledging that the healthcare professional has personal goals and motivations that conflict with professional obligations) and that the conflict is ethically impermissible (because we are acknowledging the professional’s personal goals and motivations prevent them from fulfilling their professional obligations). It also means allowing the conflict of interest to take place and to affect professional conduct when we do not allow the same to happen in the case of FCOIs. This differential treatment is not ethically justified or so we have argued.

This does not mean that any person with conscientious beliefs or values —that is, deeply held and self-identifying moral views—should be prevented from accessing a medical profession. Everyone (with the exception of some pathological cases) has conscientious beliefs and values and it is very likely that at least some of those beliefs would at least sometimes conflict with professional obligations. NFCOI, unlike most FCOI, in healthcare is probably unavoidable. What we can and should avoid is certain NFCOIs affecting patient care and proper healthcare delivery more generally. While we have not discussed what we can do to prevent any kind of NFCOI affecting patient care, we have argued that conscientious objection is actively opposing this goal by promoting involvement of the healthcare professional’s NFCOI in the professional care. If we really are concerned about conflict of interest in healthcare, we cannot ignore this implication. People should be prevented from acting on their moral beliefs when these conflict with professional obligations. While, admittedly, it can be difficult to police all interests of conscience, at the very least we should not institutionalize NFCOI in the way that conscientious objection in medicine policies currently seeks to do.

## Footnotes

1 Instead doctors who fundamentally oppose the agreed professional best practice have two options: 1. The doctor can provide the agreed medical service regardless of his or her own views. Even in the case of capital punishment, which is clearly not in the patient’s best interests, there might be reasons to offer the service as a professional duty if (i) refusal is not going to make a difference as to whether it goes ahead, (ii) the procedure will be better performed by the doctor in question than by some non-doctor, and (iii) and the prisoner requests the doctor to perform this intervention to reduce suffering. 2 The doctor can remove themselves from the profession or the professional role (costly conscientious objection). Just as the homeopathic doctor could remove himself from medical practice and set up a private homeopathy clinic (provided claims were made in line with advertising standards), a doctor who comes to believe that the agreed standards of medical ethics are in fact unethical may withdraw from the profession or take up a role within the profession where the doctor’s own beliefs do not in fact come into conflict with the agreed best practice.

2 However, other contested medical services (such as examining patients of the opposite sex (Strickland 2012) or providing vaccination) are not covered. It is not easy to explain or justify this discriminatory treatment (Giubilini 2014 and 2016). Personal ethical views about abortion, vaccination, inspecting patients of the opposite sex, and so on, can all conflict with professional obligations. It is hard to tell what grants some of these views a special status. What we have said about abortion applies equally to other practices to which a healthcare professional might have a conscientious objection, whether or not they belong to the narrow group of practices where conscientious objection is normally allowed. Muslim doctors who would want to object to medically inspecting patients of the opposite sex for religious reasons, for example, have an interest that conflicts with their professional obligations, such as the obligation to treat all their patients equally and fairly, as well as with other basic requirements of non-discrimination that characterize liberal societies.
